# Miscellany

**Published:** 1906-01

**Authors:** 


### MISCELLANY.

---

The Department of Health of Buffalo has issued the following circular: Beginning January 1, 1906, this department will add the Widal test of the blood, in suspected cases of typhoid fever, to its laboratory work. A specially designated Widal outfit will be used, and such outfits can be obtained at any Police Precinct Station House in the city.

After being used, Widal outfits must be returned, either by being delivered to the department direct or they may be sent by mail. Widal outfits are under no circumstances to be sent back to the police stations.

The directions, which will accompany each Widal outfit, fully explains what may be expected of the test.

WALTER D. GREENE, Commissioner of Health.

---

STATE Civil Service examinations for positions in the Erie County service are to be held in Buffalo, February 10, 1906. Applications must be filed in the office of the State Civil Service Commission at Albany before noon of February 6th. The positions for which the open examinations will be held are as follows:

Elevator conductor, City and County Hall; a keeper, Erie County Penitentiary or jail; matron, county institutions; orderly, Erie County Hospital; pupil nurse, and superintendent of nurses, Erie County Hospital.

